# A micropeptide regulates seed desiccation

**DOI:** 10.3389/fpls.2025.1550190

**Published:** 2025-03-26

**Authors:** Rong Lu, Zilin Zhang, Songping Hu, Hailin Xia, Huibin Han

**Affiliations:** Research Center of Plant Functional Genes and Tissue Culture Technology, College of Bioscience and Bioengineering, Jiangxi Agricultural University, Nanchang, China

**Keywords:** microRPG1, kernel dehydration rate, ethylene, mechanized harvesting, maize

## 1 Introduction

**Figure 1:**
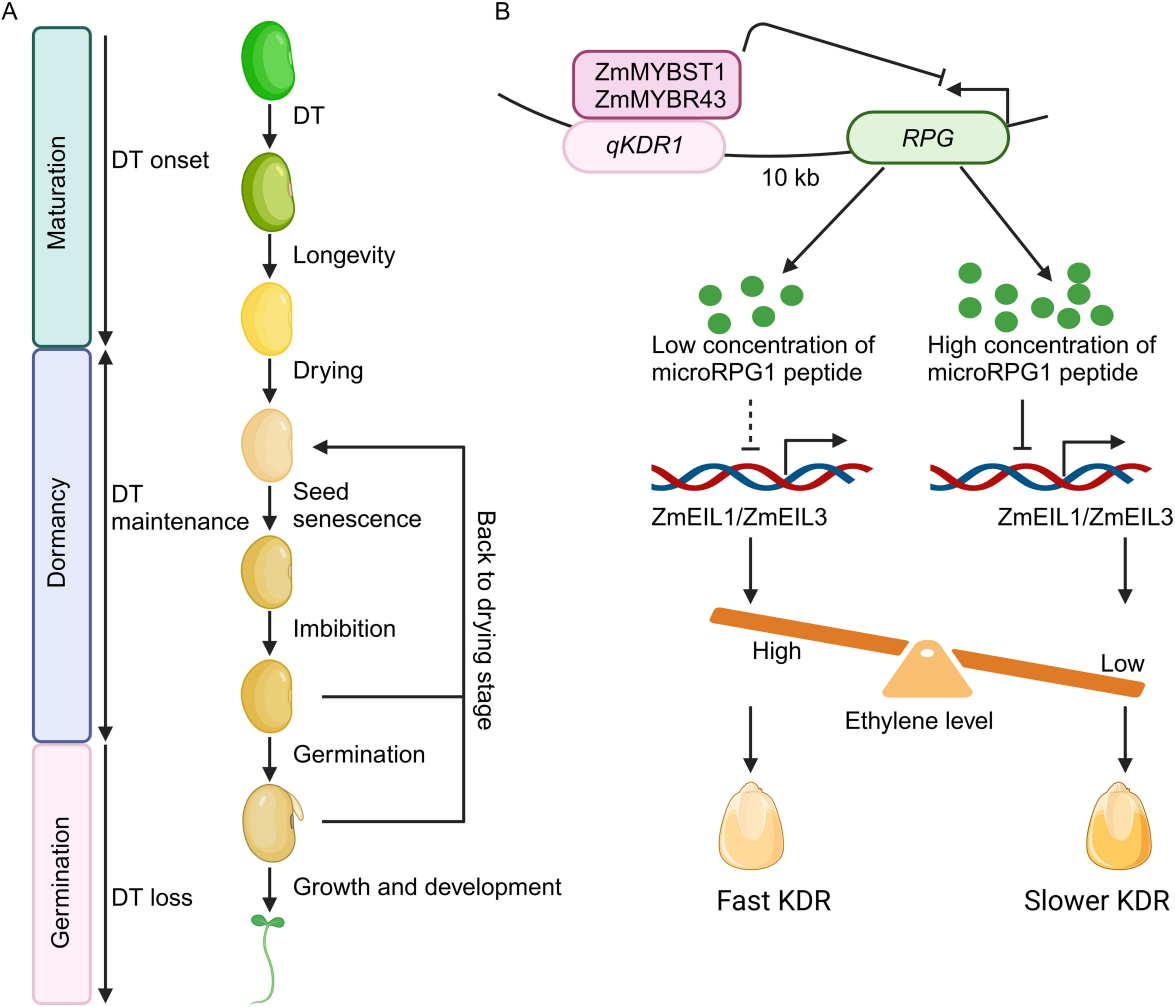
The microRPG1 peptide modulates seed desiccation through ethylene signaling pathways. **(A)** Key developmental phases in seed life. The establishment of desiccation tolerance (DT) occurs during the late maturation phase, subsequently followed by developmental processes that promote longevity during the dormancy period. Maturation drying leads to a reduction in seed moisture levels. DT is maintained by intricate networks during dormancy. Optimal conditions of low temperature and humidity prolong seed viability, whereas less favorable environmental conditions contribute to seed senescence. Water imbibition triggers metabolic activities and cellular processes, culminating in germination. Cutting-edge technologies rehydrate seeds, followed by desiccation, to enhance cellular repair and boost seed vigor. DT is lost when seeds progress to germination. **(B)** Mechanistic model of microRPG1 peptide in seed desiccation. *RPG* encodes a peptide, namely microRPG1, comprising 31 amino acids. Two MYB transcription factors, ZmMYBSt1 and ZmMYBR43, interact with the *qKDR1* locus, thereby repressing the transcriptional activity of *RPG* and the levels of microRPG1 peptide. The microRPG1 peptide subsequently regulates the expression of *ZmEIL1* and *ZmEIL3*, pivotal transcription factors in the ethylene signaling cascade, thereby modulating ethylene signaling and KDR. Elevated ethylene concentrations facilitate KDR, while reduced ethylene levels deaccelerate KDR. The figure is adapted from Yu et al., 2024. Dashed line means weak effect. The figure is created via biorender.com.

Seeds serve as the major means of reproduction for most plant species and form the foundation of both agriculture and natural ecosystems (Waterworth et al., 2024). Seeds are also the key genetic resources to deal with the increasing human population and climate fluctuations (Leprince et al., 2017). Seed development can be categorized into three major stages: maturation, dormancy, and germination (**Figure 1A**; Zinsmeister et al., 2020; Powell, 2022; Nadarajan et al., 2023; Waterworth et al., 2024). In the maturation phase, seeds acquire desiccation tolerance, followed by developmental processes that expands longevity to dormancy stage. Maturation drying reduces seed moisture content to 5% – 15% of fresh weight (**Figure 1A**; Zinsmeister et al., 2020). Dormancy of seeds under optimal conditions, such as low temperature and humidity, prolongs viability, while suboptimal conditions lead to seed aging (**Figure 1A**; Powell, 2022; Nadarajan et al., 2023). Seed dormancy is modulated by a complex interplay of genetic, biochemical, and molecular determinants intricately connected to environmental signals such as light, temperature, nitrate availability, and phytohormones including abscisic acid (ABA) and gibberellin (GA) (Chahtane et al., 2017; Matilla, 2024; Rachappanavar, 2025). The difference between dormant seeds and non-dormant seeds could be attributed to a number of gene expression changes (Meimoun et al., 2014), physiological, developmental, and morphological features of the grains on the spike, including pericarp color, transparency, hairiness, waxiness, permeability of water, α-amylase activity, and concentrations of growth regulators such as ABA and GA within the embryo (Sohn et al., 2021). Seed germination is initiated by water uptake (imbibition), resulting in activation of multiple cellular actions, and is completed with the emergence of the young roots and shoots (**Figure 1A**; Carrera-Castaño et al., 2020; Waterworth et al., 2024).

The acquisition of desiccation tolerance at the late seed maturation stage provides a critical survival mechanism for crops, enabling them to adaptive to adverse environmental conditions such as extreme temperatures and drought (Leprince et al., 2017; Zinsmeister et al., 2020; Waterworth et al., 2024). The majority of crop plants can generate seeds classified as orthodox seeds, which possess the ability to withstand drying to low moisture content (below 7%) and harsh extreme environmental conditions such as freezing (-10°C) for a long time (Nadarajan et al., 2023; Waterworth et al., 2024). In maize, the moisture content of kernel suitable for mechanized harvesting is from 15% - 25%, however, in some regions such as China, maize varieties have high grain water content at harvest, ranging from 30% - 40% (Xiang et al., 2012; Kebebe et al., 2015; Dai et al., 2017; Li et al., 2018a, 2018b; Zhou et al., 2018). Kernel dehydration rate (KDR), defined as the rate of moisture loss between two adjacent periods after pollination (Zhang et al., 2024), is a critical determinant of maize seed quality and exerts a significant impact on the efficiency of mechanical harvesting (Li et al., 2018b). Besides, the removal of free water leads to a phase transition as the cytoplasm reduces mobility from a fluid to glassy state (Buitink and Leprince, 2008), resulting in metabolic quiescence and increased seed longevity (Zinsmeister et al., 2020). To date, genetic elements implicated in the modulation of reactive oxygen species (ROS) signaling, lipid peroxidation at the cell membrane, the preservation of DNA and RNA integrity, DNA methylation status, biosynthesis of seed storage proteins (SSPs), and phytohormones such as ABA, auxin, GA, and brassinosteroids (BRs) have been documented as crucial regulators of seed longevity (Nadarajan et al., 2023; Pirredda et al., 2023; Waterworth et al., 2024). Abiotic factors including light, thermal conditions, drought and salinity stress also significantly impact seed longevity, with temperature and water availability emerging as predominant factors (Zinsmeister et al., 2020). In maize, several quantitative trait loci (QTLs) have been characterized as pivotal players in the regulation of KDR (Li et al., 2020, 2021a; Zhang et al., 2023; Jin et al., 2024). Collectively, a higher level of desiccation tolerance is crucial for maize mechanized harvesting, preventing grain breakage, mildew, and reducing the costs associated with harvest and storage (de Jager et al., 2004; Xiang et al., 2012; Kebebe et al., 2015; Wang et al., 2022; Xia et al., 2024). Thus, a comprehensive understanding of the mechanisms governing desiccation tolerance of seeds is necessary and crucial.

Seed dehydration is linked to a multitude of physiological modifications, including the accumulation of macromolecules (proteins, lipids, and carbohydrates), enhanced membrane integrity, and activation of cellular dehydration defense mechanisms, which are governed by hormone signaling pathways such as abscisic acid (ABA) and ethylene (Angelovici et al., 2010; Bewley et al., 2013; Kijak and Ratajczak, 2020; Oliver et al., 2020; Smolikova et al., 2020). The onset of desiccation tolerance occurs when seeds enter into dormancy stage at the late maturation stage (Leprince et al., 2017; Smolikova et al., 2020). Numerous signaling components including Late Embryogenesis Abundant (LEA) proteins, small heat shock proteins (sHSPs), non-reducing oligosaccharides, antioxidants, reactive oxygen species (ROS), as well as gibberellin (GA), and ABA, have been identified as crucial regulators of seed desiccation tolerance (Angelovici et al., 2010; Kijak and Ratajczak, 2020; Smolikova et al., 2020; Waterworth et al., 2024). In addition, many transcription factors such as ABA-INSENSITIVE 3 (ABI3), FUSCA 3 (FUS3) and LEAFY COTYLEDONS 2 (LEC2) have been discovered to defines the balance between GA and ABA to finally initiate the onset of seed desiccation tolerance (Smolikova et al., 2020). However, the regulatory mechanisms of seed desiccation tolerance mediated by the small signaling peptides remain largely elusive.

Micropeptides, also referred to as microproteins or short open reading frame (sORF)-encoded peptides, are essential products derived from a larger polypeptide or from MicroRNAs (miRNAs), long non-coding RNA (lncRNA), and circular RNA (circRNA), typically characterized by an arbitrary length of less than 100 - 150 amino acids (Hashimoto et al., 2008; Makarewich and Olson, 2017; Sousa and Farkas, 2018; Vitorino et al., 2021; Pan et al., 2022; Sruthi et al., 2022; Bhar and Roy, 2023; Gautam et al., 2023). A growing number of evidence show the key roles of micropeptides in various plant developmental and adaptive processes including but not limited to plant growth (Sharma et al., 2020; Erokhina et al., 2021; Badola et al., 2022), adventitious root formation (Chen et al., 2020), nodule formation (Couzigou et al., 2016), cold response (Chen et al., 2022), anthocyanin biosynthesis (Vale et al., 2024), and responses to cadmium and arsenic stressors (Kumar et al., 2023; Lu et al., 2024), and immunity (Zhou et al., 2022). Recently, the microRPG1 (micropeptide of RPG ORF1) peptide that governs kernel dehydration rate (KDR) in maize has been identified, offering novel perspectives on the molecular mechanisms that regulate seed desiccation mediated by micropeptide and providing valuable insights for future genetic breeding of cereal crops (**Figure 1B**; Lyu, 2024; Yu et al., 2024).

## 2 microRPG1 peptide regulates ethylene signaling to control maize seeds desiccation

Maize (*Zea mays*) is one of the most important crops world-wide, with an annual global production of over 1147 million tons (Yang and Yan, 2021). Mechanized harvesting of maize kernels is a viable solution to reduce labor costs and to enhance production efficiency. However, mechanized harvesting has not yet been achieved in China due to the absence of appropriate corn cultivars (Li et al., 2018a; Wang et al., 2018; Liu et al., 2020). Mechanized harvesting of maize requires a sufficiently low moisture content of kernels (15% - 25%) (Liu et al., 2020). This poses a significant challenge as the majority of corn cultivars in China exhibit a high grain moisture content during harvest, usually between 30% and 40% (Dai et al., 2017; Li et al., 2018a; Zhou et al., 2018). Consequently, enhancing KDR and minimizing kernel moisture content at the harvest stage are critical and has become a major aim of modern maize breeding (Sala et al., 2006; Qu et al., 2022). To this end, a prominent quantitative trait locus (QTL) for KDR, designated as *Kernel Dehydration Rate 1* (*qKDR1*), has been identified within the corn recombinant inbred line population, which originated from the crossbreeding of corn inbred lines K22 and DAN340, known for their variant KDRs (Pan et al., 2016; Xiao et al., 2016; Yu et al., 2024).

*qKDR1* is located on chromosome 1, specifically within a 1417 base pair (bp) intergenic non-coding region of the maize genome (Yu et al., 2024). Targeted deletion of this sequence via CRISPR-Cas9 at this locus yields varying KDRs, demonstrating that the 1417-bp segment of *qKDR1* is crucial for KDR variability, as its knockout leads to impaired KDR. To investigate the regulatory mechanism of *qKDR1* on KDR, transient transcriptional activity assays were conducted in maize protoplasts. The findings reveal that *qKDR1* functions as a silencer, with the 369-bp segment of *qKDR1* identified as the major repressive element. Subsequent RNA-sequencing analysis is performed to ascertain potential targets of *qKDR1*, leading to the identification of the target gene, *qKDR1 Regulated Peptide Gene* (*RPG*). *RPG* is situated 10 kilobases upstream of *qKDR1* and exhibits high expression levels in maize kernels, and its transcriptional activity declines during the later stages of kernel maturation. In maize lines where *qKDR1* has been knocked out, *RPG* expression is markedly elevated. Collectively, these results indicate that *qKDR1* acts as a repressor of *RPG* expression. Furthermore, analysis of public chromatin immunoprecipitation sequencing (ChIP-seq) datasets has uncovered two MYB-related transcription factors, *ZmMYBST1* and *ZmMYBR43*, that bind to the *qKDR1* locus. Both *ZmMYBST1* and *ZmMYBR43* exhibit expression patterns that similar to *RPG*, and they also inhibit *RPG* transcriptional activity. Additionally, CRISPR-Cas9-generated double mutants of *ZmMYBST1* and *ZmMYBR43* demonstrate a reduced rate of KDR. These findings suggest that *ZmMYBST1* and *ZmMYBR43* interact with the *qKDR1* region to downregulate *RPG* expression, thereby modulating KDR.

Ribosome profiling sequencing (Ribo-seq) reveals that mRNA of *RPG* is ribosome bound in three open reading frames, ORF1, ORF2, and ORF3. Mutations in *ORF1* accelerated KDR, whereas mutations in the two other ORFs has no obvious effect on KDR. Overexpressing *ORF1* resulted in a decelerated KDR. Furthermore, the kernel moisture content of *ORF1* knockout lines is decreased under different environments. The endogenous ORF1 micropeptide is also verified by immunoprecipitation (IP) and mass spectrometry (MS). These findings indicate that ORF1 encodes the functional RPG micropeptide (microRPG1). Furthermore, ZmEIL1 and ZmEIL3, key players in ethylene signaling, are identified as the downstream targets of microRPG1 peptide via RNA-seq assay. *ZmEIL1* and *ZmEIL3* are upregulated in the *microRPG1* knockout and downregulated in the overexpression lines, respectively. Consistently, *ZmEIL1* and *ZmEIL3* knockout lines also exhibit decelerated KDR. In contrast, application of ethylene facilitates KDR rate. Hence, microRPG1peptide represses ethylene signaling, which further decelerates kernel dehydration (**Figure 1B**).

## 3 Future perspectives

Although the essential function of the microRPG1 peptide in the modulation of desiccation tolerance of seed has been established in both maize and Arabidopsis (Yu et al., 2024), the precise molecular mechanisms warrant further exploration. First, the binding affinities and sites of ZmMYBST1 and ZmMYBR43 to *qKDR1* remain to be elucidated. Secondly, the mechanism by which *qKDR1* inhibits *RPG* expression, potentially through the native promoter of *RPG*, requires further investigation. It has been proposed that microRPG1 is localized at the plasma membrane, nucleus, and cytoplasm, indicating that unidentified receptors may exist and could potentially recognize the microRPG1 peptide, thereby initiating cellular signaling cascades, including ethylene signaling in the nucleus and cytoplasm to finely regulate desiccation tolerance. The advanced CRISPR screening platform provides a powerful methodology for generating single or multiple mutations of receptor-like kinases (RLKs) simultaneously (Gaillochet et al., 2021), which will facilitate the identification of uncharacterized receptors that can recognize microRPG1 signal to modulate maize KDR. Furthermore, it is plausible that the microRPG1 peptide exerts its effects independently of any specific receptors. Additionally, the interactions between the microRPG1 peptide and other phytohormones such as ABA and GA, which are implicated in the regulation of seed desiccation tolerance (Kijak and Ratajczak, 2020; Smolikova et al., 2020), necessitate further scrutiny. Importantly, single-cell transcriptomic assays have facilitated the identification of novel regulators involved in seed development (Liew et al., 2024; Yao et al., 2024), a technique that could potentially unveil the regulators of seed desiccation tolerance at a single-cell resolution and establish the unprecedented transcriptional networks mediated by microRPG1 peptide that govern seed desiccation tolerance. Notably, seeds develop desiccation tolerance during the maturation phase and sustain this tolerance during the dormancy phase (**Figure 1A**). A critical question that remains unresolved is how maize initiates the transcription and biosynthesis of the microRPG1 peptide. Furthermore, the mechanisms by which the microRPG1 peptide interacts with both known and yet-to-be-identified factors involved in seed desiccation tolerance require elucidation (Smolikova et al., 2020; Farrant et al., 2022; Waterworth et al., 2024).

Despite the fact that the microRPG1 peptide is exclusively found in the genera Zea (Yu et al., 2024), it is plausible that other yet-to-be-identified small signaling peptides may also influence desiccation tolerance. Desiccation tolerance is established during seed maturation on the maternal plant through an array of programmed cellular mechanisms (Waterworth et al., 2024), suggesting that small signaling peptides involved in the dehydration process, such as CLAVATA3/EMBRYO SURROUNDING REGION 9 (CLE9) (Zhang et al., 2019) and CLE25/26 (Takahashi et al., 2018; Endo and Fukuda, 2024), C-TERMINALLY ENCODED PEPTIDE 5 (CEP5) (Smith et al., 2020), and RAPID ALKALINIZATION FACTOR (RALF) (Jing et al., 2024), along with other drought-responsive small signaling peptides (Xie et al., 2022; Ji et al., in press; Zhang et al., 2025), could also potentially modulate seed desiccation tolerance, but this requires further examinations. Additionally, the mass spectrometry imaging (MSI) technique has been employed in in plants to elucidate the spatial distribution of structurally diverse plant hormones (Chen et al., 2024) and various other plant compounds (García-Rojas et al., 2024; Yin et al., 2024; Zou et al., 2025) even at the single-cell resolution (Croslow et al., 2024; Zhang et al., 2024). This technique has been successfully performed to identify small peptides mammalian cells (David et al., 2018; Bottomley et al., 2024). Thus, MSI could be instrumental in discovering novel small signaling peptides associated with desiccation tolerance during the late maturation phase of seeds, thereby enhancing the existing knowledge of the mechanisms underlying seed dehydration (**Figure 1A**). In addition to the microRPG1 peptide, multiple miPEPs have been discovered in various crop and horticultural species (Ji et al., in press); however, their biological roles remain largely uncharacterized. The CRISPR-Cas system can facilitate the generation of *miPEP* knockout mutants (Li et al., 2021b), and to identify potential receptors (Gaillochet et al., 2021). Moreover, CRISPR-mediated gene regulation tools, such as CRISPR interference (CRISPRi), CRISPR activation (CRISPRa), CRISPRoff, CROP-seq, CRISP-seq, CRISPR-based epigenetic modifications, and Perturb-seq (Liu et al., 2022), coupled with single-cell transcriptomics (Liew et al., 2024; Yao et al., 2024), can be utilized to elucidate the influence of miPEPs on growth, agronomic and horticultural traits, and stress response mechanisms at single cell resolution. These tools also enable the construction of novel transcriptional networks modulated by miPEP peptides.

In summary, the discovery of microRPG1 peptide contributes to understanding seed desiccation and to the improvement of corn seeds to adapt to mechanized harvesting. According to Worldostats (https:://worldostats.com/corn-maize-production-by-country-2025/) and FAO (Food and Agriculture Organization of the United Nations) (Liu et al., 2025), the global production of corn is a staggering 1.16 billion tones per year. The top 3 leading maize producing countries are the USA (348.8 million tons), China (277.2 million tons) and Brazil (109.4 million tons), accounting for over half of global maize production. The application of microRPG1 peptide would lower the moisture content of maize, and prevent grain breakage, mildew, reduce labor costs and increase maize production worldwide for food supplement in future. In addition, it is possible to introduce the *RPG* gene in to other cereal crops such as rice, wheat and millet artificially or application of exogenous microRPG1 peptide to manipulate the moisture content of seeds, which is beneficial for storage and mechanized harvesting in future. Identifying the uncharacterized signaling components and novel small signaling peptides involved in seed desiccation would provide a new genetic toolbox for the genetic enhancement of cereal crops and broaden the applications of small signaling peptides in modern agriculture.
